# Control beliefs and risk for 4-year mortality in older adults: a prospective cohort study

**DOI:** 10.1186/s12877-016-0390-3

**Published:** 2017-01-11

**Authors:** Wei Duan-Porter, Susan Nicole Hastings, Brian Neelon, Courtney Harold Van Houtven

**Affiliations:** 1 Minneapolis VA Health Services Research and Development, One Veterans Dr, Minneapolis, MN 55417 USA; 2 Durham VA Health Services Research and Development, Durham, NC USA; 3 Durham VA Geriatrics Resarch, Education, and Clinical Center, Durham, NC USA; 4 Department of Medicine, Division of Geriatrics, Duke University School of Medicine, Durham, NC USA; 5 Duke University Center for the Study of Aging and Human Development, Durham, NC USA; 6 Department of Public Health Sciences,Medical University of South Carolina, Charleston, SC USA

**Keywords:** Control beliefs, Mortality risk, Biomedical predictors

## Abstract

**Background:**

Control beliefs are important psychological factors that likely contribute to heterogeneity in health outcomes for older adults. We evaluated whether control beliefs are associated with risk for 4-year mortality, after accounting for established “classic” biomedical risk factors. We also determined if an enhanced risk model with control beliefs improved identification of individuals with low vs. high mortality risk.

**Methods:**

We used nationally representative data from the Health and Retirement Study (2006–2012) for adults 50 years or older in 2006 (*n* = 7313) or 2008 (*n* = 6301). We assessed baseline perceived global control (measured as 2 dimensions—“constraints” and “mastery”), and health-specific control. We also obtained baseline data for 12 established biomedical risk factors of 4-year mortality: age, sex, 4 medical conditions (diabetes mellitus, cancer, lung disease and heart failure), body mass index less than 25 kg/m^2^, smoking, and 4 functional difficulties (with bathing, managing finances, walking several blocks and pushing or pulling heavy objects). Deaths within 4 years of follow-up were determined through interviews with respondents’ family and the National Death Index.

**Results:**

After accounting for classic biomedical risk factors, perceived constraints were significantly associated with higher mortality risk (third quartile scores odds ratio [OR] 1.37, 95% CI 1.03–1.81; fourth quartile scores OR 1.45, 95% CI, 1.09–1.92), while health-specific control was significantly associated with lower risk (OR 0.69–0.78 for scores above first quartile). Higher perceived mastery scores were not consistently associated with decreased risk. The enhanced model with control beliefs found an additional 3.5% of participants (*n* = 222) with low predicted risk of 4-year mortality (i.e., 4% or less); observed mortality for these individuals was 1.8% during follow-up. Compared with participants predicted to have low mortality risk only by the classic biomedical model, individuals identified by only the enhanced model were older, had higher educational status, higher income, and higher prevalence of diabetes mellitus and cancer.

**Conclusion:**

Control beliefs were significantly associated with risk for 4-year mortality; accounting for these factors improved identification of low-risk individuals. More work is needed to determine how assessment of control beliefs could enable targeting of clinical interventions to support at-risk older adults.

## Background

Aging is accompanied by increasing physical limitations, more chronic health conditions, and higher risk for mortality, but there is substantial variation in individual risk for these adverse health outcomes [1–4]. Furthermore, the relative importance of biomedical, psychological, and socioeconomic risk factors for predicting outcomes, such as mortality, remains unclear. Among psychological factors thought to impact health, control beliefs have been highlighted as relevant for both health behaviors [5–7] and outcomes, including mortality [8–12].

Control beliefs refer to an individual’s perception of his/her ability to impact life events, and range from global beliefs to situationally specific perceptions [8]. Differences in control beliefs likely reflect dispositional tendencies, along with lifetime disparities in socioeconomic factors and experiences of discrimination [8–11]. Among older adults, control beliefs may affect adoption of adaptive and healthy behaviors in the face of increasing physical and cognitive limitations [7]. Furthermore, control beliefs have been associated with ability to manage certain chronic health conditions [5, 6], which contribute to mortality risk. Finally, control beliefs are associated with general perceptions of well-being and such positive psychological perceptions may be associated with decreased activation of stress and inflammatory pathways, leading to improved long-term health [6, 7, 12]. Thus, control beliefs are informed by diverse psychosocial factors and may impact risk for mortality through a variety of behavioral and physiologic pathways.

Despite some previous work suggesting the importance of control beliefs for mortality [13–17], it remains unclear if these psychological perceptions would remain relevant for mortality risk when specific biomedical factors (e.g., health conditions and functional impairments [3, 18, 19]) are taken into account. Previous studies examining the association of control beliefs with mortality adjusted for general health status and/or count of chronic health problems, but did not consider the differential importance of individual conditions or impairments [13–17]. Understanding the relationship between control beliefs and mortality, while accounting for the differentials risks associated with various health conditions and impairments, may offer further insights into the relative importance of psychological vs. biomedical factors. Additionally, some groups did not consistently find significant associations between control beliefs and mortality risk when general health measures were included in modeling [13–16]. Thus, it remains to be seen whether control beliefs improve risk prediction when individual, well-established biomedical factors are included in statistical modeling.

We used survey data from a large nationally representative study of older Americans, in order to investigate whether control beliefs were significantly associated with 4-year mortality risk, after accounting for known “classic” biomedical risk factors [3]. Next, we determined if the addition of control belief variables improved predictive accuracy of our risk model. Finally, we evaluated how incorporation of control beliefs changed the classification of low vs. high-risk individuals.

## Methods

### Study design and sample

We used 2006–2012 data for Health and Retirement Study (HRS) participants aged 50 or older at baseline (in 2006 or 2008), with at least 4 years follow-up. HRS is an ongoing observational study of representative samples of middle-aged and older adults in the United States. Biennial surveys assessed a range of demographic, economic, and health-related topics, while psychosocial surveys were given every 4 years, providing information on personality traits, control beliefs, and social support, among other factors [20, 21]. Psychosocial surveys were first given to a random 50% of participants in 2006, and to the other half first in 2008.

For determining whether control beliefs are associated with mortality risk, we used data for individuals with baseline health and psychosocial information in 2006 (*n* = 7313), called the “2006 cohort.” To evaluate accuracy of risk models and compare low vs. high-risk classification, we selected individuals with baseline data in 2008 (*n* = 6301), called the “2008 cohort.” Follow-up data were available for 98.6% and 97.6% of the 2006 and 2008 cohorts, respectively.

This study was approved by the Durham Veterans Affairs Medical Center Institutional Review Board.

### 4-year mortality outcome

We determined death by 2011 for the 2006 cohort, to account for the fact that some of the 2006 cohort were actually first interviewed in early 2007. Similarly, we assessed for death by 2013 for the 2008 cohort. Year of death was assessed by HRS exit interviews with respondents’ family. HRS actively monitored and recorded participant deaths through the National Death Index and sought out interviews with surviving family and/or partners, resulting in a completion rate of 93% for exit interviews after deaths [22].

### Classic biomedical risk factors

Using 1998–2002 HRS data for adults 50 and older, Lee et al. [3] developed and validated a multivariable logistic model for 4-year mortality. Logistic regression produces similar risk estimates as Cox proportional hazards modeling when few individuals would be censored and the outcome is relatively rare during follow-up [23]. Additionally, because logistic regression can be used to estimate risk for a given individual, it is very commonly employed to develop risk calculators for clinical settings [23, 24]. Lee et al. [3] intended to generate a model that would aid clinical risk stratification, as well as inform epidemiologic studies. They evaluated over 40 demographic, health and functional status variables, before selecting 12 risk factors: age, sex, 4 medical conditions (diabetes mellitus, cancer, lung disease and heart failure), body mass index (BMI) less than 25 kg/m^2^, current smoking, and 4 functional difficulties (with bathing, managing finances, walking several blocks and pushing or pulling heavy objects). Although developed to predict risk of death within 4 years, this model also accurately predicted 10-year mortality [25].

### Control belief variables

We assessed global control beliefs as two variables, perceived constraints and mastery, in addition to health-specific control. Perceived constraints and mastery indicate perceived external restrictions and personal competence, respectively [8, 9, 20]. Perceived constraints scores were the mean of responses on a Likert scale (1 = strongly disagree, 6 = strongly agree) to 5 statements related to feelings of helplessness and lack of control: 1) “I often feel helpless in dealing with the problems of life;” 2) “Other people determine most of what I can and cannot do;” 3) “What happens in my life is often beyond my control;” 4) “I have little control over the things that happen to me;” and 5) “There is really no way I can solve the problems I have.” Perceived mastery was the mean of responses to 5 statements indicating confidence in one’s skills and ability to act: 1) “I can do just about anything I really set my mind to;” 2) “When I really want to do something, I usually find a way to succeed at it;” 3) “Whether or not I am able to get what I want is in my own hands;” 4) “What happens to me in the future mostly depends on me;” and 5) “I can do the things that I want to do.” In support of the conceptualization of perceived constraints and mastery as two distinct dimensions, previous work has established that these items load onto two latent factors [9, 26]. Cronbach alphas of 0.86 and 0.89 have been reported for the perceived constraints and mastery scales, respectively [27, 28]. Health-specific control was assessed with “Using a 0 to 10 scale where 0 means ‘no control at all’ and 10 means ‘very much control,’ how would you rate the amount of control you have over your health these days?”

Because all control belief variables showed highly skewed distributions, we were concerned with possible nonlinear effects throughout the full range of scores. Thus, we categorized scores into quartiles to improve interpretation of associations between control beliefs and mortality risk.

### Covariates and descriptive characteristics

Per Lee et al. [3], we categorized baseline age as 50–59 years, 5-year intervals for 60–84 years, and 85 and older. We also assessed sex, race, Hispanic ethnicity, marital status, education, total household income, and health insurance status.

We used responses to questions beginning with “Has a doctor ever told you that you have …” for diabetes mellitus, hypertension, heart disease, heart failure, lung disease, cancer (“excluding minor skin cancer”), psychiatric problems and arthritis. We calculated BMI from measured height and weight, and per Lee et al. [3], we dichotomized BMI as less than 25 kg/m^2^or not. Lee et al. [3] also examined the effect of including multiple categories of BMI, but found no improved predictive performance over use of a binary variable.

We determined if respondents had memory problems, urinary incontinence, difficulty with various basic or instrumental activities of daily living (e.g., walking across room, bathing, using the telephone, and managing finances), and other functional difficulties (e.g., walking several blocks and climbing stairs). For all functional measures, questions began with “Because of a health problem do you have any difficulty…” We coded responses of “Yes” and “Can’t Do” as affirmative and “No” as negative. We encoded as missing those participants who answered “Don’t Do” or refused to answer.

We also assessed smoking at baseline, and identified those who participated in vigorous or moderate activity more than once a week.

### Statistical analysis

In order to examine whether control beliefs contributed to 4-year mortality risk after accounting for established biomedical risk factors, we first used 2006 cohort data to: 1) recalibrate the multivariable logistic model reported by Lee et al. [3]; and 2) estimate an enhanced model with control beliefs in addition to all predictors in the Lee et al. model. We provide adjusted odds ratios (OR) and 95% confidence intervals (CI) for both models. In order to avoid optimistic bias that occurs with evaluation of model performance in the same dataset used for model fitting [24, 29], we used 2008 cohort data for external validation of the classic biomedical model and the enhanced model with control beliefs (i.e., evaluated the predictive accuracy of both models in a dataset independent of the one used for fitting the models). We calculated two indicators of overall accuracy: 1) *c*-statistic describes the ability to discriminate between those who have an event and those who do not, and 2) Hosmer-Lemeshow goodness-of-fit *χ* ^*2*^ evaluates model calibration or “fit”, showing how accurately the model predicts the actual observed risk (non-significant *p*-values of 0.05 or greater indicate adequate fit).

Finally, we also used 2008 cohort data to investigate whether our enhanced risk model improved the classification of individuals into low vs. high-risk groups. We defined “low risk” as 4% or lower predicted risk; 4% was the observed mortality reported by Lee et al. [3] for those at or below median risk, and other mortality models have found similar results [30]. We calculated categorical Net Reclassification Improvement (NRI); overall NRI is the sum of event and non-event NRI, which are proportions of individuals appropriately reclassified among those with and without the outcome of interest, respectively [31, 32]. Larger NRI indicates improved classification. We also determined Cohen’s kappa statistic and McNemar’s *χ* ^2^, to examine agreement and discrepancy between the 2 models.

To address missing data, we used multiple imputations by chained equations [33] to generate pooled parameter estimates based on 25 imputed datasets, under a missingness at random assumption. Highest missingness was observed for BMI (15% in 2006 cohort, 12% in 2008 cohort); all other predictors had less than 5% missingness. Analyses were performed using R v. 3.0.2 and “predictABEL” package [34].

## Results

Baseline characteristics and outcomes for 2006 and 2008 cohorts appear in Table 1. As expected, the 2008 cohort was slightly older (mean age 69.5 years compared to 68.3 years for 2006 cohort), with concomitant small increases in the prevalence of most medical conditions and urinary incontinence. Observed 4-year mortality was nearly 12% for both cohorts.

**Table 1:** Baseline characteristics and 4-year mortality for Health and Retirement Study cohorts

Characteristics	2006 Cohort^a^ (*n* = 7313)	2008 Cohort^a^ (*n* = 6301)
Sociodemographics, % (n)		
Age in years:		
50–59	23.1 (1692)	19.0 (1198)
60–64	14.0 (1027)	12.7 (802)
65–69	19.6 (1432)	19.2 (1212)
70–74	16.4 (1200)	18.6 (1174)
75–79	12.0 (879)	14.1 (888)
80–84	8.2 (601)	8.8 (557)
≥ 85	6.6 (482)	7.5 (471)
Female	58.3 (4260)	59.9 (3773)
Race:		
White	84.4 (6175)	84.1 (5301)
Black	13.1 (960)	13.2 (833)
Hispanic	8.0 (586)	9.0 (564)
Married	64.7 (4735)	61.0 (3843)
Education:		
High school degree	32.3 (2362)	31.9 (2007)
College or above	44.1 (3224)	43.5 (2743)
Total household income^b^:		
1^st^ quartile	22.9 (1676)	23.4 (1477)
2^nd^ quartile	24.6 (1800)	26.9 (1693)
3^rd^ quartile	25.6 (1872)	25.2 (1585)
4^th^ quartile	26.9 (1965)	24.5 (1546)
Health conditions, % (n)		
Diabetes mellitus	20.3 (1487)	21.5 (1353)
Hypertension	57.4 (4195)	60.9 (3839)
Heart disease	25.1 (1837)	27.1 (1705)
Heart failure	3.5 (258)	3.8 (241)
Stroke	6.3 (461)	7.4 (467)
Cancer	15.3 (1119)	16.0 (1009)
Lung disease	10.2 (746)	11.7 (738)
Arthritis	61.2 (4479)	64.5 (4065)
Psychiatric problems	15.9 (1162)	17.4 (1098)
Body mass index <25 kg/m^2^	21.1 (1541)	21.3 (1341)
Psychological characteristics, mean (SD)		
Constraints, range 1–6	2.24 (1.19)	2.22 (1.21)
Mastery, range 1–6	4.75 (1.11)	4.77 (1.10)
Health-specific control, range 0–10	7.21 (2.39)	7.27 (2.06)
Functional impairments, % (n)		
Problems with memory	3.0 (221)	3.1 (195)
Urinary incontinence	21.4 (1565)	25.4 (1599)
Difficulty with ADL^c^:		
Walking across room	5.9 (434)	6.3 (396)
Bathing	5.2 (378)	5.5 (345)
Dressing	8.9 (652)	8.5 (533)
Eating	2.4 (174)	2.3 (142)
Transferring in or out of bed	5.3 (389)	5.0 (316)
Toileting	5.4 (395)	4.6 (287)
Difficulty with IADL^c^:		
Using the telephone	2.7 (199)	2.9 (182)
Managing finances	4.1 (301)	4.0 (255)
Shopping for groceries	7.7 (565)	7.5 (471)
Managing medications	2.7 (195)	2.6 (164)
Preparing meals	4.5 (327)	4.4 (277)
Other functional difficulties^c^:		
Walking several blocks	29.7 (2174)	30.6 (1931)
Climbing stairs	43.5 (3183)	43.4 (2734)
Pushing or pulling large object	25.0 (1825)	24.0 (1515)
Getting up from chair	41.5 (3032)	41.8 (2636)
Health behaviors, % (n)		
Vigorous activity more than once per week	23.0 (1680)	23.6 (1484)
Moderate activity more than once per week	55.8 (4078)	53.1 (3348)
Current smoking	12.8 (938)	12.7 (802)
Events, % (n)		
Deaths^d^	11.7 (856)	11.8 (743)

^a^Health and Retirement Study participants with baseline data in 2006 or 2008

^b^Quartiles assigned based on distribution of incomes from all individuals who responded in 2006 or 2008

^c^All functional limitations (including ADL = basic activities of daily living and IADL = instrumental activities of daily living) were defined using questions beginning with “Because of a health problem do you have any difficulty…” Response categories included “Yes” and “Can’t Do” (both coded as affirmative), “No” (coded as negative), and “Don’t Do” and “Refused” (both coded as missing)

^d^Death by 2011 or 2013, for 2006 or 2008 cohorts, respectively

### Association of control beliefs with 4-year mortality risk

When we refit the classic biomedical risk model for 4-year mortality [3], all risk factors were significantly associated with increased risk (Table 2), except for difficulty with pushing or pulling large objects (OR 1.21, 95% CI 1.00–1.47). In agreement with Lee et al. [3], highest risks were associated with older age categories (reference 50–59 years), with OR increasing steadily through 85 and older, although the relative increases in risk were slightly lower than those previously reported (Table 2). Factors associated with the next highest levels of increased risk were having heart failure (OR 2.43, 95% CI 1.81–3.27), difficulty with bathing (OR 2.41, 95% CI 1.62–2.84), and difficulty with walking several blocks (OR 2.11, 95% CI 1.75–2.56; Table 2).

**Table 2:** Risk factors for 4-year mortality in multi-variable logistic modeling

Risk factors	Classic model^a^		Enhanced model with control beliefs^b^	
	Adjusted OR (95% CI)	*p*-value	Adjusted OR (95% CI)	*p*-value
Demographics				
Age in years:				
50–59	1	―	1	―
60–64	1.61 (1.09–2.36)	0.016	1.66 (1.13–2.45)	0.010
65–69	2.57 (1.85–3.57)	<0.001	2.68 (1.92–3.73)	<0.001
70–74	3.10 (2.22–4.34)	<0.001	3.22 (2.30–4.50)	<0.001
75–79	4.44 (3.17–6.21)	<0.001	4.64 (3.31–6.51)	<0.001
80–84	6.65 (4.71–9.39)	<0.001	6.74 (4.76–9.53)	<0.001
≥ 85	14.4 (10.2–20.4)	<0.001	14.5 (10.2–20.5)	<0.001
Male	1.84 (1.56–2.17)	<0.001	1.85 (1.57–2.18)	<0.001
Health conditions & behaviors				
Diabetes mellitus	1.64(1.37–1.97)	<0.001	1.63 (1.36–1.97)	<0.001
Heart failure	2.43 (1.81–3.27)	<0.001	2.37 (1.76–3.19)	<0.001
Cancer	1.54 (1.27–1.86)	<0.001	1.52 (1.26–1.84)	<0.001
Lung disease	1.61 (1.29–2.00)	<0.001	1.58 (1.27–1.97)	<0.001
BMI <25 kg/m^2^	1.69 (1.40–2.05)	<0.001	1.70 (1.41–2.06)	<0.001
Current smoker	1.89 (1.50–2.39)	<0.001	1.83 (1.45–2.31)	<0.001
Functional impairments				
Bathing	2.14 (1.62–2.84)	<0.001	2.00 (1.51–2.64)	<0.001
Managing finances	1.40 (1.02–1.93)	0.035	1.28 (0.93–1.76)	0.136
Walking several blocks	2.11 (1.75–2.56)	<0.001	1.97 (1.62–2.39)	<0.001
Push/pulling large object	1.21 (1.00–1.47)	0.056	1.12 (0.92–1.37)	0.252
Control beliefs				
Perceived constraints^c^:				
1^st^ Quartile	―	―	1	―
2^nd^ Quartile	―	―	1.14 (0.86–1.51)	0.365
3^rd^ Quartile	―	―	1.37 (1.03–1.81)	0.029
4^th^ Quartile	―	―	1.45 (1.09–1.92)	0.010
Perceived mastery^c^:				
1^st^ Quartile	―	―	1	―
2^nd^ Quartile	―	―	0.79 (0.62–0.99)	0.039
3^rd^ Quartile	―	―	0.86 (0.68–1.09)	0.200
4^th^ Quartile	―	―	0.95 (0.74–1.22)	0.691
Health-specific control^c^:				
1^st^ Quartile	―	―	1	―
2^nd^ Quartile	―	―	0.72 (0.57–0.91)	0.006
3^rd^ Quartile	―	―	0.69 (0.54–0.89)	0.004
4^th^ Quartile	―	―	0.78 (0.61–0.99)	0.010

OR = odds ratio; 95% CI = 95% confidence interval; BMI = body mass index

^a^Includes all risk factors from model developed by Lee et al. [3]

^b^Includes all risk factors from model developed by Lee et al. [3] and 3 control belief variables (perceived constraints, perceived mastery, and health-specific control)

^c^Scores divided into quartiles, see Methods

In our enhanced model with control beliefs, we found significantly increased mortality risk associated with higher levels of perceived constraints and lower levels of health-specific control (Table 2). Perceived constraints scores in the third and fourth quartiles were associated with higher risks (OR 1.37, 95% CI 1.03–1.81 and OR 1.45, 95% CI, 1.09–1.92, respectively), whereas all scores for health-specific control above the first quartile were associated with lower risk (OR 0.69–0.78; Table 2). Perceived mastery scores in the second quartile were associated with decreased risk (OR 0.79, 95% CI 0.62–0.99), but scores in the third and fourth quartiles were not significantly associated with risk for mortality (Table 2). With the addition of control belief variables, the classic biomedical risk factors largely remained significant and of similar risk magnitude, except for difficulty with managing finances, which became non-significant (classic biomedical model OR 1.40, 95% CI 1.02–1.93, and enhanced model with control beliefs OR 1.28, 95% CI 0.93–1.76).

### Comparison of classic biomedical and enhanced risk models for 4-year mortality

We used 2008 cohort data to evaluate predictive accuracy for the classic biomedical and enhanced risk model with control beliefs. We found no difference between the two models in overall performance. For discrimination, *c*-statistics were 0.815 and 0.817 for the classic and enhanced models, respectively. The Hosmer-Lemeshow tests also showed adequate fit for both models (*χ* ^2^ *p*-value = 0.09 for classic model vs. 0.24 for enhanced model). When we compared low vs. high-risk classification by the two models, we identified significant improvement in reclassification with the enhanced model (categorical NRI 0.026, 95% CI 0.016–0.036, p-value < 0.001). Event NRI was 0.13%, indicating a relatively small proportion of individuals who died were more appropriately classified by the enhanced model. In contrast, non-event NRI was 2.41%, showing that the enhanced model more appropriately classified a larger proportion of people who remained alive during follow-up. Although there was high agreement between the 2 models (Cohen’s kappa 0.88), we also found evidence of differential categorization (McNemar’s *χ* ^2^ *p*-value <0.001).

More than a quarter of the 2008 cohort were classified as having low risk by both models, while 15.8% (*n* = 311) of these individuals were differentially categorized by the two models (Table 3). Among individuals who were differentially categorized, those who were identified as low-risk by only the enhanced model were generally older, had higher education status and household incomes, and had higher prevalence of diabetes mellitus and cancer (Table 3). Observed 4-year mortality was lowest (1.0%) among those who were predicted to have low risk by both models, followed by 1.8% for those classified as low-risk by only the enhanced model, and lastly, 5.0% for individuals identified as low-risk by only the classic model.

**Table 3:** Characteristics of individuals with low predicted 4-year mortality risk by classic vs. enhanced models

	Low risk^a^			Not low risk (*n* = 4327)
	Both models (*n* = 1663)	Only classic model (*n* = 89)	Only enhanced model (*n* = 222)	
Predicted risk, mean (SD)				
Classic Model^b^	2.3 (0.8)	3.5 (0.3)	4.7 (0.4)	17.0 (15.0)
Enhanced Model^c^	2.2 (0.8)	4.6 (0.5)	3.6 (0.3)	17.1 (15.3)
Observed deaths, % (n)	1.0 (17)	5.0 (5)	1.8 (4)	16.6 (717)
Sociodemographics, % (n)				
Age in years:				
50–59	55.2 (918)	33.7 (30)	12.2 (27)	5.2 (223)
60–64	23.1 (384)	33.7 (30)	22.1 (49)	7.8 (339)
65–69	12.9 (214)	7.9 (7)	51.4 (114)	20.3 (877)
70–74	8.8 (147)	24.7 (22)	5.4 (12)	22.9 (993)
75–79	0 (0)	0 (0)	9.0 (20)	20.1 (868)
80–84	0 (0)	0 (0)	0 (0)	12.8 (556)
≥ 85	0 (0)	0 (0)	0 (0)	10.9 (461)
Female	76.8 (1278)	65.2 (58)	68.0 (151)	52.8 (2286)
Race:				
White	82.3 (1369)	85.4 (76)	85.6 (190)	84.7 (3666)
Black	13.5 (225)	7.9 (7)	13.5 (30)	13.2 (571)
Other	4.1 (69)	6.7 (6)	0.9 (2)	2.1 (90)
Hispanic	11.7 (195)	16.9 (15)	6.8 (15)	7.8 (339)
Married	70.2 (1168)	61.8 (55)	64.0 (142)	56.4 (2440)
Education:				
High school degree	29.8 (496)	32.6 (29)	28.8 (64)	32.8 (1418)
College or above	55.7 (926)	37.1 (33)	54.5 (121)	38.4 (1663)
Total household income^d^:				
1^st^ quartile	12.6 (210)	22.5 (20)	16.2 (36)	28.0 (1211)
2^nd^ quartile	18.3 (304)	28.1 (25)	27.9 (62)	30.1 (1302)
3^rd^ quartile	27.6 (459)	22.5 (20)	20.7 (46)	24.5 (1060)
4^th^ quartile	41.5 (690)	27.0 (24)	35.1 (78)	17.4 (754)
Has health insurance	90.5 (1505)	88.8 (79)	95.9 (213)	97.8 (4231)
Health conditions, % (n)				
Diabetes mellitus	6.9 (115)	9.0 (8)	18.9 (42)	27.5 (1188)
Hypertension	46.8 (778)	58.4 (52)	57.7 (128)	66.6 (2881)
Heart disease	10.3 (171)	18.0 (16)	20.7 (46)	34.0 (1472)
Heart failure	0 (0)	0 (0)	0 (0)	5.6 (241)
Stroke	2.0 (33)	2.2 (2)	2.7 (6)	8.9 (386)
Cancer	4.3 (71)	3.4 (3)	17.6 (39)	20.7 (895)
Lung disease	1.4 (24)	10.1 (9)	5.9 (13)	16.0 (692)
Arthritis	50.4 (838)	62.9 (56)	65.8 (146)	69.9 (3025)
Psychiatric problems	16.5 (16.5)	24.7 (22)	14.9 (33)	17.7 (768)
BMI <25 kg/m^2^	12.5 (208)	12.4 (11)	18.9 (42)	25.0 (1080)
Functional impairments, n (%)				
Problems with memory	1.1 (18)	0 (0)	0.5 (1)	4.1 (176)
Urinary incontinence	21.3 (354)	27.0 (24)	20.7 (46)	27.2 (1175)
Difficulty with ADL:				
Walking across room	0.5 (8)	0 (0)	0.5 (1)	8.9 (387)
Bathing	0 (0)	0 (0)	0 (0)	8.0 (345)
Dressing	1.7 (28)	5.6 (5)	1.4 (3)	11.5 (497)
Eating	0 (0)	0 (0)	0.5 (1)	3.3 (141)
Transferring in or out of bed	1.1 (19)	4.5 (4)	3.2 (7)	6.6 (286)
Toileting	0.7 (11)	2.2 (2)	0.9 (2)	6.3 (272)
Difficulty with IADL:				
Using the telephone	0.4 (6)	3.4 (3)	0 (0)	4.0 (171)
Managing finances	0.5 (8)	1.1 (1)	1.4 (3)	5.6 (243)
Shopping for groceries	0.7 (12)	3.4 (3)	1.8 (4)	10.4 (452)
Managing medications	0.6 (10)	4.5 (4)	0.9 (2)	3.4 (148)
Preparing meals	0.2 (3)	2.2 (2)	0.9 (2)	6.2 (270)
Other functional difficulties:				
Walking several blocks	3.8 (63)	13.5 (12)	15.3 (34)	42.1 (1822)
Climbing stairs	29.3 (487)	42.7 (38)	27.9 (62)	47.4 (2049)
Push or pull large object	27.0 (449)	41.6 (37)	34.2 (76)	50.2 (2172)
Getting up from chair	10.8 (180)	23.6 (21)	12.2 (27)	29.7 (1287)
Health behaviors, % (n)				
Vigorous activity^e^	29.2 (485)	13.5 (12)	23.0 (51)	17.2 (743)
Moderate activity^e^	53.2 (884)	42.7 (38)	53.2 (118)	37.3 (1614)
Current smoker	7.5 (124)	16.9 (15)	9.9 (22)	14.8 (641)

*ADL*= basic activities of daily living, *IADL*= instrumental activities of daily living, *SD* = standard deviation, *BMI*= body mass index

^a^Defined as predicted 4-year mortality risk of 4% or less

^b^Includes all risk factors from model developed by Lee et al. [3]

^c^Includes all risk factors from model developed by Lee et al. [3], and control belief variables in quartiles (perceived constraints, perceived mastery, and health-specific control)

^d^Quartiles based on distribution of incomes from all individuals who responded in 2008

^e^Level of activity as specified, occurring more than once a week

## Discussion

Using data from a nationally representative sample of older Americans, we evaluated an enhanced model for 4-year mortality risk that accounted for control beliefs and a set of classic biomedical risk factors [3]. In our enhanced model, high scores for perceived constraints were significantly associated with increased mortality risk, and high scores for health-specific control were associated with decreased risk. The impact of perceived mastery was less clear, with significantly decreased risk only for second quartile scores. Although both classic and enhanced models performed similarly in overall predictive accuracy, we found that inclusion of control beliefs improved classification of individuals into low vs. high-risk groups.

In refitting the classic 4-year mortality risk model by Lee et al. [3], we found similar risks associated with most of the original predictors, although several had smaller magnitudes of increased risks. Our sample was somewhat older (e.g., 23% of 2006 cohort were 50–59 and 20% were 65–69, compared with 27% and 15% for those age groups reported by Lee et al. [3]). Additionally, Lee et al. [3] used geographic location to select the sample for model development, thus excluding participants from the southern United States; we used nationally representative data at all stages of our analysis.

Our enhanced model separately accounted for specific important biomedical risk factors and demonstrated that perceived constraints and health-specific control were independently associated with meaningful differences in mortality risk. Past studies examining the relationship between control beliefs and mortality assessed health as a single self-reported general rating and/or a count of medical conditions [13–17]; therefore, they failed to adjust for differential risks associated with individual conditions or disabilities [3, 18]. Furthermore, some of this previous work found no significant association between control beliefs and mortality, when general measures of health were incorporated into risk models [15, 16]. Thus, our results contribute to the evidence supporting the importance of control beliefs for mortality risk.

It is unclear why perceived mastery was not as important for mortality risk in our analysis. Past studies have suggested that “positive” beliefs may be less relevant than “negative” control perceptions for health and physical function [9, 26, 35]. Possibly, respondents may be more accurate in their assessments of constraints compared with mastery, particularly with respect to socioeconomic factors. Or, perceived constraints may have greater impact on health-related behaviors or physiologic pathways. Further work will be required to understand the relative roles and consequences of positive vs. negative control beliefs, as well as global vs. health-specific perceptions.

Accounting for control beliefs may be especially important for predicting mortality risk in older individuals who have one or more serious medical diagnoses. Our enhanced model differentially classified 3.5% of individuals as low-risk; the observed mortality in this group was less than 2%, well below our 4% threshold for low risk. In this group, 65.8% were 65 or older, while nearly a fifth had diabetes and 17.6% had cancer diagnoses [5, 6]. In contrast, those classified as low-risk by only the classic model had an observed mortality of 5%, and comparatively, had lower income and education status. Socioeconomic factors, such as education, perceived social status, and perceived discrimination, have been associated with variation in control beliefs [9–11, 17]. Some have found that control beliefs may moderate the impact of educational status [17], while others have reported that perceived control may mediate the relationship between socioeconomic status and health outcomes [36]. Thus, future work is needed to better understand the interplay between control beliefs and socioeconomic factors, and whether the impact of control beliefs in our enhanced model may be due, in part, to known relationships between socioeconomic factors and health.

Our results indicate that accounting for control beliefs could improve risk stratification of older adults. Mortality risk models are being increasingly used for diverse purposes, including selection of appropriate preventative services [37, 38] and adjusting goals for chronic disease management [39]. For example, routine colorectal cancer screening is not recommended for adults aged 76–85 [40], but some in this age range may have low mortality risk and thus, have time to benefit from continued screening [41]. Additionally, we may be able to address disparities in general control beliefs with targeted services and support, particularly for disadvantaged groups. Control beliefs reflect diverse psychosocial factors, including dispositional traits, socioeconomic characteristics, and life experiences, offering us an important opportunity to integrate diverse patient factors and improve personalization of clinical care.

There are some limitations to our study. Logistic regression does not readily account for the length of exposure to risk factors, and estimates from such models may diverge from those using survival analyses, such as Cox proportional hazards functions, over a longer period of follow-up, for more frequent outcomes, and/or with substantial censoring (e.g., due to incomplete follow-up) [23]. In our study, mortality was uncommon within the 4-year timeframe and follow-up was excellent; thus, logistic modeling is likely comparable to survival methods in estimating mortality risk for this period. We used self-reported data for health conditions and functional impairments; self-reported health information generally correspond well with data from medical records, but may be biased towards under-reporting [42, 43]. Depressive symptoms were not examined in the current analyses because depressive symptoms were not included as predictors in the original biomedical model validated by Lee et al. [3] In previous work, we found low baseline prevalence of depressive symptoms in the HRS cohort and no significant relationship between these symptoms and risk for poor health outcomes [44]; however, depressive symptoms may be an important area for future study in other samples. We also did not determine the influence of other individual conditions or specific combinations of medical diagnoses; these may be important to explore in future work. The HRS cohort also has high education status and low functional impairments; thus, our results may not be generalizable to those with poorer mental health, lower education, and/or greater impairments. Finally, in this non-experimental study, there remains the possibility of unmeasured confounders contributing to associations between control beliefs and mortality risk.

## Conclusions

In summary, our results indicate that control beliefs are important for predicting mortality risk in older adults. Moreover, control beliefs may account for risks derived from diverse psychosocial sources and enable implementation of targeted interventions. Future work is needed to determine how assessment of control beliefs may be incorporated into clinical care and whether we can better support those at increased risk due to high perceived constraints and/or low health-specific control.

## Abbreviations

95% CI: 95% Confidence Interval
BMI: Body mass index
HRS: Health and retirement study
NRI: Net reclassification improvement
OR: Odds ratio
